# Investigating Nutrition-Related Complications and Quality of Life in Patients With Gastroenteropancreatic Neuroendocrine Tumors: Protocol for a Mixed-Methods Prospective Study

**DOI:** 10.2196/11228

**Published:** 2018-12-19

**Authors:** Erin Laing, Nicole Kiss, Michael Michael, Karla Gough, Meinir Krishnasamy

**Affiliations:** 1 Department of Nursing University of Melbourne Melbourne Australia; 2 Nutrition and Dietetics Department Peter MacCallum Cancer Centre Melbourne Australia; 3 Institute of Physical Activity and Nutrition School of Exercise and Nutrition Sciences Deakin University Geelong Australia; 4 Department of Cancer Experiences Research Peter MacCallum Cancer Centre Melbourne Australia; 5 Division of Cancer Medicine & Neuroendocrine Unit Peter MacCallum Cancer Centre Melbourne Australia; 6 Victorian Comprehensive Cancer Centre Melbourne Australia

**Keywords:** neuroendocrine tumor, diet, food, and nutrition, malnutrition, diet, vitamins, signs and symptoms, niacin, patient experience

## Abstract

**Background:**

Gastroenteropancreatic neuroendocrine tumors (GEP NETs) are a heterogeneous group of tumors with distinct effects on the body due to their potential to secrete hormones and peptides. The incidence and prevalence of GEP NETs in Australia are rising. During 2000-2006, the annual incidence was approximately 3.3 per 100,000 population. To date, there has been development of clinical practice and consensus guidelines for NETs covering best practice for diagnosis, treatment, and medical management; however, the supportive care needs and optimal nutritional management of patients affected by NETs remains underresearched, and evidence to guide clinical practice is lacking. While there is emerging research describing the extent of morbidity in different types of GEP NET patients, little is known about the experience of people affected by these tumors and how nutritional status is impacted by either diagnosis or treatment.

**Objective:**

The objective of this study was to explore nutrition-related complications and quality of life of patients diagnosed with a GEP NET and to generate evidence to inform future research and development of nutrition screening and management practices.

**Methods:**

Patients diagnosed with a GEP NET at two metropolitan recruitment sites will be invited to participate in a 6-month, mixed-methods longitudinal study. Participants recruited to the study will receive usual care and participate in data collection for the study at 4 time points (at recruitment and 2, 4, and 6 months postrecruitment). Study data will include nutritional status, body weight, fat-free mass, and patient-reported outcome measures (dietitian contact, disease-related symptom presence and severity, dietary habits, health-related quality of life, psychological morbidity, and financial impact). At recruitment and 6 months postrecruitment, complete nutrient testing, including relevant plasma vitamin levels, will also be undertaken. A purposive sample of participants will be invited to take part in semistructured interviews to explore the experience of living with a GEP NET and associated nutrition complications.

**Results:**

Ethics approval has been obtained, and study recruitment and data collection are underway.

**Conclusions:**

This study will provide the first in-depth, comprehensive description of nutritional issues in patients with GEP NETs. Results will advance the knowledge of nutritional issues faced by patients with GEP NETs and help inform the development of screening tools and clinical practice guidelines.

**International Registered Report Identifier (IRRID):**

DERR1-10.2196/11228

## Introduction

Neuroendocrine tumors (NETs) are a heterogeneous group of tumors most commonly located in the gastrointestinal (GI) tract, lung, and pancreas and are characterized by their propensity to secrete hormones and peptides. Gastroenteropancreatic (GEP) NETs arise in the neuroendocrine cells of the GI tract, most commonly the gastric mucosa, small or large intestine, and pancreas [1]. GEP NETs account for around 60% of all diagnosed cases and are considered a rare and complex disease that requires high-level multidisciplinary consultation and care [2]. GEP NETs have increased in incidence and prevalence over the past two decades. During 2000-2006, the annual incidence of GEP NETs in Australia was estimated to be 3.3 per 100,000 population [3]. In the United States alone, GEP NETs affect approximately 170,000 people, and their prevalence is greater than gastric, pancreatic, esophageal, or hepatobiliary adenocarcinomas [2,4].

Patients with GEP NETs can experience numerous and complex symptoms relating to the tumor mass effect (from the primary site or metastases), generalized symptoms of malignancy, side effects of hormonal hypersecretion, or treatment-related side effects [2,5]. Up to 30% of GEP NET patients, particularly those with tumors located in the small bowel, have carcinoid syndrome whereby their tumors secrete endogenous amine hormones, which can give rise to symptoms including flushing, fatigue, severe diarrhea, food intolerance, restlessness, fluctuations in mood, and pain [1,5]. GEP NET-related symptoms may persist for long periods (median 9.2 years) prior to diagnosis and, thus, have potential to substantially impact an individual’s quality of life and health care service utilization [6-9]. The potential side effects of the disease and its treatment have been well documented [6,10]. Nevertheless, there is very limited published information on the nutritional impact of this disease and its treatment on patients.

Some studies have demonstrated decreased health-related quality of life (HRQoL) in patients with NETs compared with that in a healthy population [6,11-14]. However, there is a paucity of Australian data and limited information on the relationship between dietary changes, nutritional issues, and HRQoL. In the first global survey that examined the effects of an NET diagnosis on 1928 NET participants, most patients (71%) reported that an NET diagnosis had a substantially negative impact on their personal life, including reduced energy levels and reduced ability to perform household chores. Over half (58%) reported making dietary changes as a result of their NET [8]. To date, only 2 small studies have explored the presence of dietary change in patients diagnosed with an NET, and details on the extent of dietary change among NET patients are yet to be explored [15,16].

There is limited published information on the nutritional impact of GEP NETs. The severity and number of symptoms and side effects arising from carcinoid syndrome and its treatments are likely to have a major impact on a patient’s ability to consume an adequate diet and have the potential to lead to inadequate nutrient intake, weight loss, and malnutrition. Malnutrition rates for GI cancers overall are well documented; however, there are limited data or published rates of malnutrition among patients with GEP NETs. There are 2 recent studies that have indicated that as many as 1 in 4 NET patients are malnourished, as assessed using the Malnutrition Universal Screening Tool and Subjective Global Assessment (SGA) tool [17,18]. Malnutrition has substantial negative consequences for cancer patients including increased mortality, poorer quality of life, increased health care costs, and reduced ability to cope with the demands of treatment [19,20].

Research is now emerging on the impact of serotonin-producing NETs, or treatment of NETs with somatostatin analogues, on niacin (vitamin B3) deficiency and risk of pellagra [21,22]. Pellagra is a nutritional deficiency characterized by dermatitis, diarrhea, and mental disturbance, which can lead to death in severe cases if untreated [21,23]. Evidence has indicated that the prevalence of biochemical or subclinical niacin deficiency may be as high as 30%-45%, but only 2 studies so far have reported on this [21,22]. More research is required to understand the relationship between NET tumors and niacin deficiency in order to inform screening practices and potential treatment options. There has also been some indication in the literature that up to 80% of patients with NETs are at risk for fat-soluble vitamin deficiency due to functional symptoms and resulting fat-malabsorption [15,24]; however, these studies were small and limited to cross-sectional data. The relationship between fat-soluble vitamin deficiency, NET diagnosis, and treatment needs further exploration to determine the extent of this risk and whether it needs to be addressed.

Although guidelines have been developed regarding the management of GEP NETs, they focus on the diagnostics and aspects of medical management of the disease but lack evidence-based information regarding nutritional management [1,25]. There is strong evidence to support early and intensive nutrition intervention in other GI cancers [19,26]; however, nutrition research to date has not focused on NETs. This reflects their rarity, heterogeneous nature, and the complex specialist care they require.

This study aims to provide detailed information on the nutritional impact of NETs on people affected by these complex tumors. Study findings will generate evidence to inform future research focused on the development of a nutrition screening and intervention program. The main objective of this study is to describe issues related to nutrition and HRQoL during diagnosis and treatment of a GEP NET including the following: (1) the prevalence of objectively assessed malnutrition and vitamin deficiencies and (2) the prevalence and severity of patient-reported physical symptoms, anxiety and depressive symptomatology, financial burden, and patient-reported dietary habits.

## Methods

### Methodology

This mixed-methods, prospective longitudinal study will be performed at 2 metropolitan sites in Melbourne, Australia. The study will be conducted by staff at Peter MacCallum Cancer Centre and the University of Melbourne, Australia. Ethics approval has been obtained from the Peter MacCallum Cancer Centre Human Research Ethics Committee (EC00235) on April 20, 2017; the study will be conducted according to the National Health and Medical Research Council’s National Statement on Ethical Conduct in Human Research 2007 (and updates) and the World Medical Association Declaration of Helsinki 2013. The study is being undertaken in part-fulfillment of a PhD at the University of Melbourne.

### Study Population

Patients attending their initial appointment at upper GI and NET clinics at participating sites will be approached to participate in the study. Eligible patients will be identified through screening of clinic lists and discussion with the NET multidisciplinary team at recruitment sites. Eligible participants will be those with a confirmed diagnosis of GEP NET, aged ≥18 years, receiving treatment or medical care at the recruitment site, able to communicate in English independently or having an interpreter present, and medically fit to participate (as per self or clinician report). Patients will be eligible for recruitment if they are within 6 weeks of their initial clinic visit because decisions regarding diagnosis and treatment that impact their eligibility are often made within that period. Patients will be ineligible if their NET is under observation only and they are, therefore, not required to attend regular appointments or if their care is transferred to another hospital not involved in the study. Both recruitment and data collection will occur in the outpatient setting when participants are attending for usual care. As this study is being undertaken as part of a PhD, the same researcher (PhD candidate) will perform recruitment and data collection at both participating sites.

**Figure 1:**
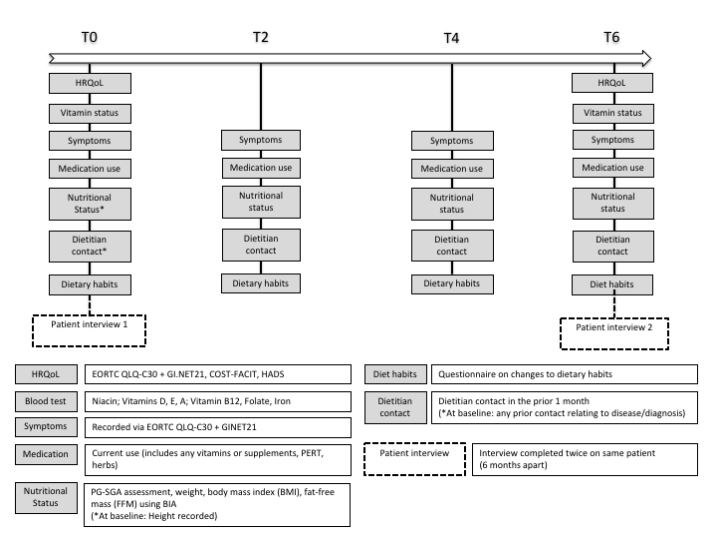
Data collection map. HRQoL: health-related quality of life; EORTC QLQ-C30+GI.NET21: European Organisation for Research and Treatment of Cancer Quality of Life Questionnaire-C30+module GI.NET21; COST-FACIT: Comprehensive Score for Financial Toxicity‐Functional Assessment of Chronic Illness Therapy; HADS: Hospital and Anxiety Depression Scale; PG-SGA: Patient-Generated Subjective Global Assessment; BIA: bioelectrical impedance analysis; PERT: pancreatic enzyme replacement therapy.

Participation will involve a 6-month data collection period from the time of recruitment, and data will be collected at 4 time points: recruitment or baseline (T0), 2 months postrecruitment (T2), 4 months postrecruitment (T4), and 6 months postrecruitment (T6; Multimedia Appendix 1 and Figure 1). If participants are admitted to an inpatient ward during their participation in the study, they will remain in the study unless deemed too unwell to do so by the coordinating principal investigator and their treating medical practitioner.

### Patient and Treatment Characteristics

Participant demographics will be collected from the medical records at baseline including age (in years), sex, ethnicity, date of diagnosis, tumor site, tumor grade and classification, treatment received (currently or previously received), and comorbidities present. Participants will be classified into categories based on demographic, disease, and treatment measures collected from the medical history (Textbox 1). Further patient-reported information will be collected at baseline using a customized self-report questionnaire including marital status, education level, employment status, and length of time experiencing symptoms prior to baseline. In this questionnaire, information on any previous nutrition- or diet-related diagnosis (eg, irritable bowel syndrome, food intolerance or allergy, diabetes, and inflammatory disease) will also be collected as presence of these conditions has the potential to confound results.

### Assessments and Data Collection

#### Health-Related Quality of Life and Symptoms

Participants will complete the European Organisation for Research and Treatment of Cancer Quality of Life Questionnaire (EORTC QLQ)-C30 and the NET-specific EORTC QLQ module GI.NET21 at all time points (T0, T2, T4, and T6) to determine HRQoL symptom prevalence and severity. The EORTC QLQ-C30 is a 30-item questionnaire composed of multi-item scales and single items, including 5 functional scales, 3 symptom scales, and 1 global health and quality of life scale [27]. The EORTC QLQ module GI.NET21 is designed to supplement completion of the QLQ-C30 and contains a total of 21-items, including a combination of single-item assessments and scales. The combined tools contain 51 items, the majority of which have a response format regarding the past week [27]. The combined EORTC QLQ-C30 and QLQ module GI.NET21 has been validated to assess quality of life and symptoms in patients with NETs [27]. Symptom severity is measured using a 4-point Likert scale (not at all, a little, quite a bit, and very much). Symptom prevalence and severity will be calculated using scores from the 3 symptom scales of the EORTC QLQ-C30 and 3 of the symptom scales (endocrine function, GI symptoms, and treatment-related symptoms) in the GI.NET21.

At T0 and T6, participants will also complete the Comprehensive Score for Financial Toxicity‐Functional Assessment of Chronic Illness Therapy (COST-FACIT) to measure financial burden and the Hospital and Anxiety Depression Scale (HADS) to measure the presence of anxiety and depression. The COST-FACIT (version 1) contains 11-items and is one of the only available patient-reported outcome measures that describe the financial burden experienced by patients [28]. The HADS is a valid and reliable 14-item questionnaire with a self-rating 4-point Likert scale that assesses symptom severity and caseness of anxiety disorders and depression and is validated for use with adult cancer patients [29,30]. Scores from the HADS are divided into 2 subscales: depression (HADS-D) and anxiety (HADS-A), which are calculated by summation, with increasing scores indicating an increasing burden of depression and anxiety [29]. If at any stage, a participant reports elevated levels of distress or reports a score of 13 or above on the anxiety and depression subscales of the HADS, with their permission, this information will be shared with the treating team, and a referral to psychology services will be considered.

**Disease characteristics.**

**Tumor site**

- Small intestine
- Colon
- Pancreas
- Other

**Tumor grading (World Health Organization Classification 2010)**

- Neuroendocrine tumor G1
- Neuroendocrine tumor G2
- Neuroendocrine carcinoma G3

**Tumor classification**

- Serotonin-producing gastrinoma
- Insulinoma
- Glucagonoma
- Vasoactive intestinal peptide tumor
- Pancreatic polypeptide tumor
- Somatostatinoma
- Calcitoninoma
- Growth hormone-releasing hormone tumor
- Neurotensinoma
- Adrenocorticotropin hormone-secreting tumor
- Growth hormone-releasing factor tumor
- Parathyroid hormone-related peptide tumor
- Unknown

#### Nutrient Testing

Participants recruited from the lead study site will be screened twice for nutrient deficiencies, using blood (serum) and urine samples, at T0 and T6. Only the lead study site will participate in nutrient testing due to funding restrictions and the increased cost of transferring samples from multiple sites. Serum samples will be collected from participants at the lead study site pathology department and analyzed for the following: vitamin B12, folate, iron studies, vitamin D, vitamin A, and vitamin E. Vitamin K, a fat-soluble vitamin, was excluded from analysis due to the requirement for analysis at an additional interstate pathology site, which was beyond the scope and budget of this study. Participants will be asked to complete a 24-hour urine sample collection for analysis of niacin status.

#### Nutritional Status and Anthropometric Measures

Nutritional status will be measured at all time points (T0, T2, T4, and T6) to enable measurement of change over time. Measures of nutritional status will include the following: height (at T0 only), body weight, body mass index (BMI), fat-free mass (FFM; see below) using bioelectrical impedance analysis (BIA) scales, and validated Patient-Generated SGA (PG-SGA) nutrition assessment tool (see below). Means and percent change relative to baseline (T0) will be calculated for each time point.

#### Fat-Free Mass

FFM will be measured using foot-to-foot BIA, which is a safe measure of body composition and useful to complement weight and BMI data. The heels of the foot are placed on 2 plates and the toes placed on the other 2 plates while the electrical current is carried via the anterior plate, and the voltage drop is measured over the posterior electrode [31,32]. The foot-to-foot technique has been demonstrated to be safe and effective in estimating FFM in cancer patients [31,33]. BIA will be measured on a commercially available device (Tanita Inc, Tokyo, Japan; model TI SC 330S). For each participant, total FFM (kg) will be calculated at each time point, and then, the change in FFM between time points will be calculated. A clinically significant difference in FFM between time points will be defined as 0.5-1.0 kg, which is based on previous studies of cancer patients [34,35].

#### Patient-Generated Subjective Global Assessment

Nutritional status and the presence of malnutrition will be measured using the nutritional assessment tool, the scored PG-SGA [36,37]. The PG-SGA has been evaluated as suitable for use as an outcome measure in clinical nutrition studies and validated for use in oncology patients [38]. The PG-SGA demonstrates a high degree of interrater reproducibility (alpha=.64) as well as high sensitivity (98%) and specificity (82%) compared with other validated tools [38].

The scored PG-SGA consists of 2 sections: a history section completed by the patient and a clinician-completed section. Each component of the PG-SGA is assigned a score depending on the impact on nutritional status and a global assessment rating (SGA category). Change in PG-SGA score is more sensitive to subtle changes in nutritional status than in SGA category. The possible global assessment ratings in the PG-SGA are as follows: SGA A (well-nourished), SGA B (suspected or moderate malnutrition), or SGA C (severe malnutrition). Global PG-SGA score will be calculated to enable description of the prevalence of malnutrition. Therefore, participants will be classified into 1 of 2 categories: well-nourished (SGA score A) or malnourished (SGA score B or C).

#### Dietitian Contact

The contact received from a dietitian will be assessed at T0, T2, T4, and T6. Dietetic interventions received by participants during the study period will be collected from medical records where available. In addition, participants will also complete a 5-item purpose-designed questionnaire regarding contact they have had with a dietitian in the previous month. This questionnaire was created specifically for this study because a validated tool does not exist for these purposes.

#### Dietary Habits

Dietary habits of participants will be assessed at T0, T2, T4, and T6 to determine whether people diagnosed with an NET make any changes to dietary habits as a result of their disease or treatment. This 5-item questionnaire was developed specifically for the purpose of this study, as to our knowledge, a brief validated tool assessing changes in dietary habits and behaviors of cancer patients does not currently exist.

#### Medication Use

All medication usage, either prescription or nonprescription, including vitamin supplements and herbal medication will be recorded at T0, T2, T4, and T6.

#### Patient Interviews

Patient interviews will be carried out at 2 time points (T0 and T6) to gather information from patients about their experience of living with and undergoing treatment for an NET, with a focus on experience regarding nutrition-related complications and dietary change. The interviews will be semistructured. A purposively selected sample of participants who consented to take part in the study will be invited to participate in an interview. A purposive sampling method will be used to ensure a diverse representation of participant experiences as part of the interview data. Where possible, diversity will be achieved with regard to participant demographics, diagnosis, and treatment type. A maximum of 15 interviews will be completed based on evidence that data saturation (where no new themes emerge from interviews) commonly occurs after 10-12 interviews [39]. Data from participant interviews will be audiorecorded and transcribed verbatim. A thematic content analysis approach will be used to analyze interview data, which will be coded by the coordinating principal investigator and a cross-section of interviews cross-coded by supervisors to check the interrater reliability of themes identified.

### Sample Size

The sample size will be pragmatic and determined by the combined number of patients attending the upper GI and NET clinics at the 2 participating sites. A minimum recruitment target of 80 patients, over 12 months, is based on an average minimum of 110 patients diagnosed with NETs across both participating sites. Recruitment estimates were based on the recruitment activity of a research project, with similar inclusion criteria, currently running at the lead participating site.

Using the approach described by Barlett et al [40], the margin of error for estimates of means based on EORTC QLQ-C30 and GI.NET21 data range from 2.2% to 8.8% of the scale or item ranges. These calculations assumed a sample of 80 patients, an alpha level of .05, a possible range of 100 points for each scale or item, and population SDs ranging from 10 to 40 [41,42]. For binary outcomes like PG-SGA classifications (ie, well-nourished vs malnourished), 80 subjects will yield a 5% margin of error of no greater than 11%.

### Statistical Analysis

Descriptive statistics (counts and percentages, means and SDs, or medians and interquartile ranges, as appropriate) will be used to summarize patient demographic and clinical characteristics, including nutritional outcomes (nutritional status and vitamin deficiencies), and responses to patient-reported outcome measures (EORTC QLQ-C30, EORTC QLQ-GI.NET21, COST-FACIT, and HADS) at baseline and follow-up assessments. Descriptive statistics will also be used to summarize medication use and patients’ responses to customized items on surveys covering dietitian contact and dietary habits. Descriptive statistics will be used to summarize the characteristics of patients who take part in the semistructured interviews.

Patient-reported outcome measures and customized items will be compared between subgroups of patients defined by nutritional outcomes (eg, malnutrition status as determined by the PG-SGA) and disease- and treatment-related characteristics (eg, disease type and stage) at recruitment and at 6 months postrecruitment using *t* tests or analysis of variance with post hoc testing as required. Evidence-based guidelines for the interpretation of between-groups differences in EORTC QLQ-C30 scores [43] will be used to characterize the size of observed differences on functional scales, symptom scales, and single items. In the absence of evidence-based guidelines for the EORTC QLQ-GI.NET21, the Cohen *d* effect size will be calculated and used to characterize the size of observed differences and interpreted using existing conventions [44]. Specifically, effect sizes will be interpreted as follows: 0.2, small-sized difference; 0.5, medium-sized difference; and 0.8, large-sized difference. Note that in the case of 3 or more subgroups, we will select a reference group, and all effect sizes will be calculated with respect to the reference group. If a nonparametric method is required to compare patient-reported outcomes between subgroups of patients, the bootstrap percentile method will be used to examine the difference of medians [45]. The confidence level of the intervals will be set at 0.95, and the number of bootstrap replications will be set at 10,000. In the case of 3 or more subgroups, we will select a reference group and compare all other subgroups to that reference group. All data will be entered into SPSS Version 23 or higher (Chicago IL, USA), and SPSS will be used for scoring, descriptive analysis, and parametric tests.

## Results

Approval from the Peter MacCallum Cancer Centre Human Research Ethics Committee was obtained in April 2017. Study recruitment and data collection are underway.

## Discussion

Clinical practice guidelines and consensus guidelines for GEP NETs with regards to best practice for diagnosis, treatment, and medical management are available, but the supportive care needs and optimal nutritional management of patients affected by these unique tumors remain underresearched, and evidence to guide clinical practice is lacking. The pathophysiology of the disease and its treatment can cause distressing symptoms that can have significant effects on vitamin synthesis and absorption, dietary habits, weight change, and appetite; however, evidence of the nature and impact of these complications is scant. Vitamin deficiency (niacin and fat-soluble vitamins) and malnutrition may be prevalent among patients with GEP NET, but there are limited data available to guide tailored screening and intervention programs.

This study will aim to provide the first in-depth, comprehensive description of nutritional issues in patients with GEP NETs and the first Australian data on nutritional complications in patients with GEP NETs. This study is deliberately targeting patients with all types of GEP NETs to enable evaluation and comparison of various disease and treatment types. Results of this study will inform further research approaches targeting specific nutritional complications and at-risk groups, later informing the development of evidence-based nutrition screening, intervention, and management practices in this patient group.

## Appendix

Multimedia Appendix 1 Schedule of data collection.

Multimedia Appendix 2 Peer-review evidence.

## Abbreviations

BIA: bioelectrical impedance analysis
BMI: body mass index
COST-FACIT: Comprehensive Score for Financial Toxicity‐Functional Assessment of Chronic Illness Therapy
EORTC QLQ-C30: European Organisation for Research and Treatment of Cancer Quality of Life Questionnaire-C30
EORTC QLQ-GI.NET21: European Organisation for Research and Treatment of Cancer Quality of Life Questionnaire module GI.NET21
FFM: fat-free mass
GEP NET: gastroenteropancreatic neuroendocrine tumor
GI: gastrointestinal
HADS: Hospital Anxiety and Depression Scale
HRQoL: health-related quality of life
PG-SGA: Patient-Generated Subjective Global Assessment
SGA: Subjective Global Assessment
