# Supplementary material for: Investigating Nutrition-Related Complications and Quality of Life in Patients With Gastroenteropancreatic Neuroendocrine Tumors: Protocol for a Mixed-Methods Prospective Study
Source: JMIR Res Protoc. 2018 Dec 19;7(12):e11228. doi: 10.2196/11228 (PMC6315228; doi:10.2196/11228)
Supplement: Multimedia Appendix 1 [file resprot_v7i12e11228_app1.pdf]

Table 1. Schedule of data collection.

Data and measure		Method					Site		Time after initial appointment at recruitment site (months)						
		A[a]	Q[b]	U[c]	B[d]	MR[e]	PM[f]	ONJ[g]	0	1	2	3	4	5	6
HRQoL[h]	EORTC QLQ-C30[i]		X				X	X	X						X
	EORTC QLQ-GI.NET21[j]		X				X	X	X						X
	COST-FACIT[k]		X				X	X	X						X
	HADS[l]		X				X	X	X						X
Symptoms	EORTC QLQ-C30		X				X	X	X		X		X		X
	EORTC QLQ-GI.NET21		X				X	X	X		X		X		X
Vitamins and iron	Niacin			X			X		X						X
	Vitamin D				X		X		X						X
	Vitamin E				X		X		X						X
	Vitamin A				X		X		X						X
	Vitamin B12				X		X		X						X
	Folate				X		X		X						X
	Iron studies				X		X		X						X
Nutritional status	PG-SGA[m]		X				X		X		X		X		X
	BIA[n] (FFM[o])	X					X	X	X		X		X		X
	Weight	X					X	X	X		X		X		X

	Height	X			X	X	X			
	Body mass index	X			X	X	X	X	X	X
Dietitian contact	Questionnaire 1.0, Section 1		X	X	X	X	X	X	X	X
Dietary habits	Questionnaire 1.0, Section 2		X		X	X	X	X	X	X
Medication	Questionnaire 2.0		X	X	X	X	X	X	X	X
Interview	N/A[p]				X	X	X			X

[a]A: anthropometric measure.
[b]Q: questionnaire
[c]U: 24-hour urine sample.
[d]B: blood test.
[e]MR: medical record.
[f]PM: Peter MacCallum Cancer Centre.
[g]ONJ: Olivia Newton-John Cancer Wellness & Research Centre.
[h]HRQoL: health-related quality of life.
[i]EORTC QLQ-C30: European Organisation for Research and Treatment of Cancer Quality of Life Questionnaire-C30.
[j]EORTC QLQ-GI.NET21: European Organisation for Research and Treatment of Cancer Quality of Life Questionnaire Module GI.NET21
[k]COST-FACIT: Comprehensive Score for Financial Toxicity-Functional Assessment of Chronic Illness Therapy.
[l]HADS: Hospital and Anxiety Depression Scale.
[m]PG-SGA: Patient-Generated Subjective Global Assessment.
[n]BIA: bioelectrical impedance analysis.
[o]FFM: fat-free mass.
[p]N/A: not applicable.
