# Supplementary material for: Investigating Nutrition-Related Complications and Quality of Life in Patients With Gastroenteropancreatic Neuroendocrine Tumors: Protocol for a Mixed-Methods Prospective Study
Source: JMIR Res Protoc. 2018 Dec 19;7(12):e11228. doi: 10.2196/11228 (PMC6315228; doi:10.2196/11228)
Supplement: Multimedia Appendix 2 [file resprot_v7i12e11228_app2.pdf]

Application ID: ONJ16009

16th December 2016

Erin Laing
Melbourne, VIC
AUSTRALIA

Dear Erin

Thank you for your recent application for an ONJ Centre Supportive Care Research PhD Scholarship. I am pleased to advise you that your application has been successful and would like to make you a provisional offer of up to **$76,340.00 (ex-GST)** subject to the successful completion of a set of agreed milestones.

On acceptance of this offer the ONJ office will negotiate milestones with you and then put in place a funding agreement with your administering organisation.

Please be advised that the Minister for Health may wish to formally announce the recipients of this funding scheme, therefore I request that no public announcement is made by you or any staff at the relevant administering, research or clinical organisations until further advice from the ONJ Centre.

Please contact me at your earliest convenience by email or phone to confirm you wish to accept this offer, or to seek further clarification.

Yours sincerely

Julie Cairns

General Manager Ambulatory Cancer Services

Cancer & Neurosciences CSU | Olivia Newton-John Cancer & Wellness Centre

PO Box 5555 Heidelberg Vic 3084
